# Comparing autotransporter β-domain configurations for their capacity to secrete heterologous proteins to the cell surface

**DOI:** 10.1371/journal.pone.0191622

**Published:** 2018-02-07

**Authors:** Wouter S. P. Jong, Maaike Schillemans, Corinne M. ten Hagen-Jongman, Joen Luirink, Peter van Ulsen

**Affiliations:** 1 Section Molecular Microbiology, Department of Molecular Cell Biology, Amsterdam Institute for Molecules, Medicines and Systems (AIMMS), Vrije Universiteit Amsterdam, Amsterdam, The Netherlands; 2 Abera Bioscience AB, Stockholm, Sweden; Centre National de la Recherche Scientifique, Aix-Marseille Université, FRANCE

## Abstract

Monomeric autotransporters have been extensively used for export of recombinant proteins to the cell surface of Gram-negative bacteria. A bottleneck in the biosynthesis of such constructs is the passage of the outer membrane, which is facilitated by the β-domain at the C terminus of an autotransporter in conjunction with the Bam complex in the outer membrane. We have evaluated eight β-domain constructs for their capacity to secrete fused proteins to the cell surface. These constructs derive from the monomeric autotransporters Hbp, IgA protease, Ag43 and EstA and the trimeric autotransporter Hia, which all were selected because they have been previously used for secretion of recombinant proteins. We fused three different protein domains to the eight β-domain constructs, being a Myc-tag, the Hbp passenger and a nanobody or V_HH_ domain, and assessed expression, membrane insertion and surface exposure. Our results show that expression levels differed considerably between the constructs tested. The constructs that included the β-domains of Hbp and IgA protease appeared the most efficient and resulted in expression levels that were detectable on Coomassie-stained SDS-PAGE gels. The V_HH_ domain appeared the most difficult fusion partner to export, probably due to its complex immunoglobulin-like structure with a tertiary structure stabilized by an intramolecular disulfide bond. Overall, the Hbp β-domain compared favorably in exporting the fused recombinant proteins, because it showed in every instance tested a good level of expression, stable membrane insertion and clear surface exposure.

## Introduction

Monomeric autotransporters are secreted proteins of the Type V secretion pathway that have been used extensively as a versatile system to export recombinant proteins to the cell surface or medium of Gram-negative bacteria [1–3]. In nature, Gram-negative species typically use autotransporters to secrete large exoproteins like proteases, adhesins and toxins across their complex cell envelope, which consists of the inner and outer membranes separated by the peptidoglycan-containing periplasmic space. Autotransporters are multi-domain proteins that include a signal sequence at the N-terminus, a β-domain at the C-terminus and in between the secreted passenger domain that carries the function of the protein. The signal sequence interacts with the Sec translocon for transport across the inner membrane after which it is cleaved off. In the periplasm, the β-domain targets the autotransporter to the outer membrane, folds into a β-barrel structure and facilitates passenger secretion. Different autotransporters carry β-domains of different sizes and some include extracellular subdomains [1, 3–8]. However, invariably all β-domains have a structural moiety at the C-terminus, here referred to as the β-core [3, 4, 8]. It comprises the C-terminal ~300 amino acids and folds into a twelve-stranded β-barrel with an N-terminal β-helical linker segment occupying its lumen. The β-barrel fold is typical for outer membrane proteins (OMPs) and like other OMPs the autotransporter β-domain is inserted into the outer membrane with the aid of the Bam complex (for β-barrel assembly machine) [9–11]. The transport of the passenger domain across the outer membrane presumably proceeds through the barrel lumen [12] and this occurs while the β-domain is assembled into its final conformation by the Bam complex (for current secretion models see: [3,13]). After secretion, the passenger domains of most autotransporters are cleaved from their β-domains through a specific proteolytic step, after which they either remain attached to the cell surface via non-covalent interactions or are released into the extracellular milieu.

The type V secretion pathway further includes trimeric autotransporters, two-partner secretion systems and intimin-like proteins [3]. Trimeric and monomeric autotransporters share the tri-partite domain organization. However, the β-domain of trimeric autotransporters is shorter and donates four β-strands to a trimeric 12-stranded β-barrel that resembles that of monomeric β-domains [14, 15] (S1 Fig). Furthermore, the passenger domains of trimeric autotransporters are very different from monomeric autotransporter passenger domains and intertwine to form long fiber-like structures consisting of flexible stalks and bulkier and globular head regions. By contrast, most monomeric autotransporter passenger domains fold into a rigid β-helical stem with protruding subdomains as exemplified by the solved crystal structures of various autotransporters [3]. An exception are the esterases/lipases like EstA of *Pseudomonas aeruginosa*. These monomeric autotransporters have passenger domains that fold into an α-helical globular domain and are not cleaved from their β-domains [6].

**Fig 1:**
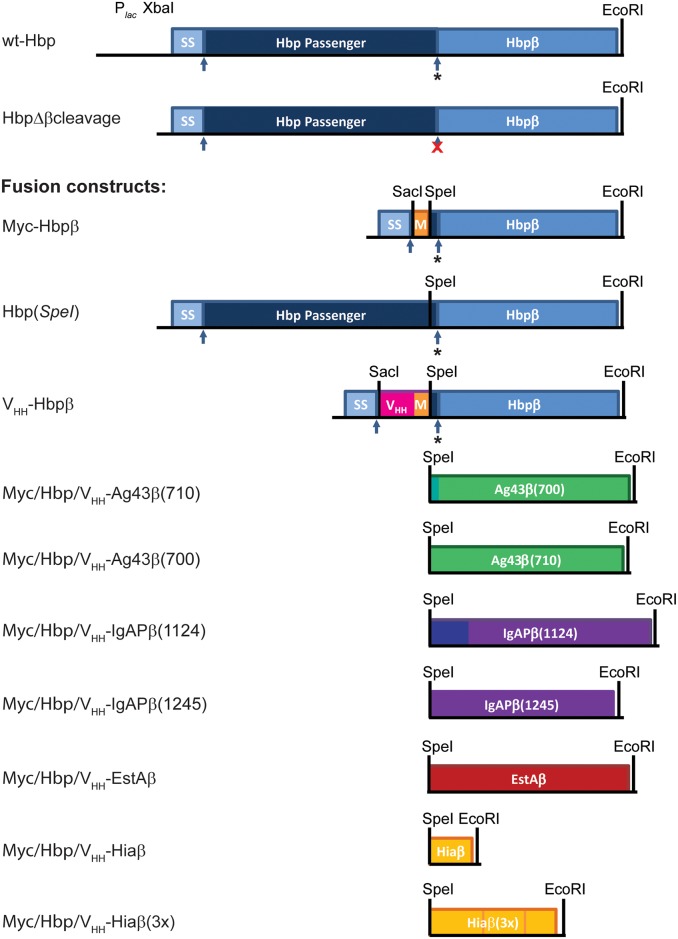
Fusion of β-domains of Hbp, Ag43, IgA protease, EstA and Hia to various passengers. Schematic representation of the Hbp andbeta-domain fusion constructs used in this study. Shown on top is the wild-type Hbp construct as cloned in plasmid pEH3. All other constructs were cloned into the same pEH3 backbone and included the Hbp signal sequence. The arrows indicate the positions of the proteolytic cleavage sites for the Hbp signal sequence (ss) and the autocatalytic cleavage to separate the passenger and β-domain at the cell surface (*). In Hbp-Δβcleavage the latter cleavage site is mutated (red **X**). The mutation results in display of the passenger from the cell surface [19, 23] and this construct served as a control for cell surface exposure of the passenger. Eight different β-domain constructs were fused to three different passengers; the Myc tag (orange), the Hbp(*SpeI*) passenger (dark blue; not drawn to scale), and the V_HH_ domain (pink). C-terminal of the V_HH_ domain a Myc tag was included (orange), followed by a Gly-Ser-Gly-Ser-Gly spacer to connect the V_HH_-Myc sequences to the respective β-domains. The eight β-domains were fused to the different passengers using a *Spe*I-*EcoR*I cassette. The β domain constructs used are Hbpβ (light blue), Ag43β(700) and Ag43β(710) (green), IgAPβ(1124) and IgAPβ(1245) (purple), EstAβ (brown), Hiaβ and Hiaβ(3x) (yellow).

We have studied the structure and secretion mechanism of the *Escherichia coli* autotransporter hemoglobin protease (Hbp), which is a member of the SPATE family (for serine protease autotransporters of *E**nterobacteriaceae*) [11, 12, 16–21]. Crystal structures are available for both the secreted passenger domain and the β-domain [18, 22]. The Hbp β-domain (Hbpβ) adopts the β-core structure described above and has no extracellular subdomains (Fig 1). The passenger domain forms a very stable β-helical stem of ~100 Å from which functional subdomains protrude that are dispensable for Hbp secretion and can be replaced by recombinant protein sequences [18, 23–26]. Furthermore, our results indicate that secretion of the Hbp passenger domain is hampered when folded segments with disulfide bonds are included [16, 17, 23, 25].

Various monomeric autotransporters have been used to secrete recombinant proteins (*e*.*g*. enzymes, peptide libraries and vaccine antigens) to the cell surface [1–3, 27, 28]. In general, the protein sequences of interest have been inserted upstream of the β-domain of the selected autotransporter, thereby replacing most of the resident passenger domain (Fig 1). However, the expression and secretion levels of specific β-domains are difficult to compare in the published studies given the different β-domains used to fuse recombinant sequences to and the different species in which these fusions were expressed, which also often differs from the species where the selected autotransporter β-domain originated from. The variation in the conditions used for expression and the assays used for analysis of protein export further complicates comparative analyses. Finally, the propensity of the fused recombinant protein to adopt a folded conformation in the periplasm also influences its expression and secretion [1, 16, 17, 25, 29, 30].

In this study, we systematically compared the suitability of five autotransporter β-domains for the export of recombinant proteins. We fused three structurally different passenger domains to eight β-domain constructs (Fig 1). The β-domain constructs derive from monomeric autotransporters Hbp and Ag43 of *E*. *coli*, IgA protease of *Neisseria gonorrhoeae*, EstA of *Pseudomonas aeruginosa* and from trimeric autotransporter Hia of *Haemophilus influenza*. Importantly, all have been used as carriers for export of recombinant proteins before [23, 25, 31–37]. They were fused to a small Myc tag, the Hbp passenger domain, and a single chain antibody (V_HH_; also referred to as nanobody [38]). The latter adopts a folded conformation prior to secretion (S1 Fig). The results showed that the different β-domain constructs showed limitations in their capacity to transport a folded passenger to the cell surface. However, in all experiments the Hbpβ constructs compared favorably to the other β-domain constructs tested here.

## Materials and methods

### Strains and growth conditions

*E*. *coli* strains TOP10F’ (Invitrogen), MC1061 [39], MC1061*degP*::S210A [40], and MC1000 derivatives DHB4 and DHBA (*dsbA*::Km) [41] were grown at 37°C in LB medium containing 0,2% glucose. The antibiotics chloramphenicol (30 μg/ml), tetracycline (12.5 μg/ml), streptomycin (30 μg/ml) and kanamycin (50 μg/ml) were added, when appropriate.

### Reagents, enzymes and sera

Restriction enzymes were from New England Biolab (NEB) or Roche Applied Science (Roche). Alkaline phosphatase & T4-DNA ligase (Rapid DNA Dephos & Ligation Kit), Lumi-Light Western blotting substrate and Proteinase K (recombinant, PCR grade) were from Roche Applied Science. Phusion High-Fidelity DNA polymerase was obtained from Thermo Scientific. All other reagents and enzymes were from Sigma-Aldrich. The rabbit polyclonal antisera against the Hbp β-domain (α-Hbpβ), the IgAPβ (α-IgAPβ), periplasmic chaperone SurA (α-SurA) and outer membrane protein OmpA (α-OmpA) have been described previously [8, 12]. The anti-myc tag rabbit antiserum ab9106 (α-Myc) was purchased from Abcam. Secondary goat-anti-rabbit antibodies conjugated to horseradish peroxidase (HRP) were purchased from Rockland.

### Plasmid construction

Plasmid pEH3 served as the backbone for all constructs made for this study [42]. The sequences of the primers used are listed in S1 Table. Fig 1 gives an overview of the constructs used in this study and the domains they include, while Table 1 gives an overview of the calculated molecular weights of the different domains.

**Table 1:** Calculated molecular weights for the constructed proteins in kDa.

	Included sequence	β-domain	ss-Myc-β	-Myc-β	-Hbp-β	-V_HH_-β
**Hbpβ**	1091–1377	30.6 *	39.4	33.6	142.4**	47.9
**Ag43β(700)**	700–1039	36.2	43.8	37.9	146.7	52.2
**Ag43β(710)**	710–1039	35.0	42.6	36.7	145.6	51.0
**IgAPβ(1124)**	1124–1532	45.1	52.6	46.8	155.6	61.1
**IgAPβ(1245)**	1245–1532	31.5	39.1	33.3	142.1	47.5
**EstAβ**	314–646	37.4	45	39.2	148	53.4
**Hiaβ**	920–1020	10.2	17.8	12	120.8	26.2
**Hiaβ(3×)**	***	21.6	29.2	23.4	132.2	37.6
**Fused Passenger**				1.8	110.7	17.3
**Hbp signal sequence**	1–52		5.8			

*Calculated for the Hbp autocleavage site

**Calculated for Hbp(*SpeI*)

***See S1 Fig

First, plasmid pEH3-Hbp(SpeI) was created, which is a derivative of pEH3-Hbp [16] that includes a *Spe*I restriction site at nucleotides 3286–3273 of the *hbp* ORF, which is the region that encodes the junction, of passenger and β-domain. The plasmid was generated using overlap-extension PCR using the mutagenesis primers Hbp SpeI fw and Hbp SpeI rv. To construct plasmids encoding a fusion between the Hbp passenger and the β-domains of Ag43 (Ag43β(700 and Ag43β(710)), IgAP (IgAPβ(1124 and IgAPβ(1245)), EstA (EstAβ) and Hia (Hiaβ), respectively, DNA fragments encoding these β-domains, including their cognate α-helical, were amplified by PCR. All fragments encoded flanking *Spe*I/*Eco*RI restriction sites. Fragments encoding Ag43β(700) and Ag43β(710) were generated using forward primers SpeI-Ag43-β-long fw and SpeI-Ag43-β-short fw, respectively. In both cases, Ag43-β rv was used as the reverse primer and *E*. *coli* MG1655 genomic DNA was used as a template. To generate IgAPβ(1124) and IgAPβ(1245) encoding fragments forward primers SpeI-IgAP-β-long fw and SpeI-IgAP-β-short fw were used in combination with primer IgAP-β rv as the reverse primer and plasmid pEN440 carrying *iga* [8] as the template. The EstA β-domain encoding fragment was amplified using *Pseudomonas aeruginosa* PAO1 genomic DNA as a template in combination with the primers SpeI-EstA-β fw and EstA-β rv. The nucleotide fragment encoding the Hia β-domain was generated using *Haemophilus Influenzae* Rd KW20 genomic DNA as a template. The primers used were SpeI-Hia-β fw and Hia-β rv. To create Hiaβ(3×) a synthetic DNA fragment encoding three interconnected Hia β-domains (including a single N-terminal α-helix) and flanking *Spe*I/*Eco*RI restriction sites was ordered from GeneArt/LifeTech. The nucleotide and amino acid (AA) sequence of this fragment can be found in S1 Fig The β-domain fragments were cloned into pEH3-Hbp(*Spe*I) using the *Spe*I and *Eco*RI restriction sites, yielding the following plasmids: pEH3-Hbp-Ag43β(700), pEH3-Hbp-Ag43β(710), pEH3-Hbp-IgAPβ(1124), pEH3-Hbp-IgAPβ(1245), pEH3-Hbp-EstAβ, pEH3-Hbp-Hiaβ and pEH3-Hbp-Hia3β, respectively.

Plasmids encoding fusions of a nanobody (V_HH_) to the Hbpβ, Ag43β(700), Ag43β(710), IgAPβ(1124), IgAPβ(1245), EstAβ, Hiaβ and Hia3β domains all used the N-terminal signal sequence of Hbp and included a myc-tag followed by a Gly-Ser-Gly-Ser-Gly spacer in between V_HH_ and the respective β-domains (Fig 1). Initially, a fragment encoding V_HH_ R2 including a C-terminal myc-tag [43] was produced by PCR using V_HH_ R2-encoding plasmid DNA (gift L. Rutten, QVQ, Utrecht, the Netherlands) as a template in combination with primers Cas/V_HH_(R2) fw and Cas/Myc rv. The resulting product was cloned into pEH3-HbpD(Δd1) [23] using the *Sac*I/*Bam*HI restriction sites in that plasmid, yielding pEH3-Hbp-V_HH_(R2). In a second PCR, this plasmid and primers pEH-XbaI-Hbp fw and V_HH_(R2)-GSGSG-SpeI rv were used to generate a *Xba*I-*Spe*I fragment encoding a fusion between the Hbp signal sequence and V_HH_ R2. This fragment was subsequently cloned into the pEH3 plasmids that encoded the Hbp passenger β-domain fusions described above to result in plasmids pV_HH_(R2)-Hbpβ, pV_HH_(R2)-Ag43(β700), pV_HH_(R2)-Ag43(β710), pV_HH_(R2)-IgAP(β1124), pV_HH_(R2)-IgAP(β1245), pV_HH_(R2)-EstAβ, pV_HH_(R2)-Hiaβ and pV_HH_(R2)-Hia3β, respectively.

To create plasmids encoding a Gly-Ser-Gly-Ser-Gly-spaced fusion between the Myc tag and the various β-domains, thereby removing the V_HH_ domain (Fig 1), a PCR was carried out using pEH3-HbpD(Δd1) as a template and the primers pEH-XbaI-Hbp fw and ssHbp-Myc-BamHI. The product was cloned into the pV_HH_(R2)-plasmid series described above yielding pSS-myc-Hbpβ, pSS-myc-Ag43(β700), pSS-myc-Ag43(β710), pSS-myc-IgAP(β1124), pSS-myc-IgAP(β1245), pSS-myc-EstAβ, pSS-myc-Hiaβ and pSS-myc-Hia3β, respectively. The constructs all contain the Hbp signal sequence followed by the Myc tag with flexible linker and then the various β-domains (Fig 1).

Nucleotide sequences of all constructs were verified by DNA sequencing (Macrogen).

### Protein expression and analysis

Recombinant proteins were expressed from vector pEH3 under control of a lacUV5 promoter [42] using the following procedure: overnight cultures were diluted to an OD_660_ of 0.05 and grown to an OD_660_ of ~0.3 to ~0.5. Cells were induced for protein production by the addition of Isopropyl β-D-1-thiogalactopyranoside (IPTG) (1 mM) or left uninduced (negative control) as indicated. Growth was continued for 2 h, after which culture samples were prepared. Cells and spent medium were separated by low speed centrifugation. Cells were directly resuspended in SDS-sample buffer (125 mM Tris–HCl, pH 6.8, 2% SDS, 10% glycerol, 0.02% bromophenol blue, 100 mM DTT) to yield whole cell lysates. Medium samples were first trichloroacetic acid (TCA)-precipitated and subsequently resuspended in an equal volume of SDS-sample buffer. All samples were heated for 5–10 min at 96°C prior to analysis by SDS-PAGE and subsequent Coomassie (G-250) staining or Western blotting. The amounts loaded of the whole-cell lysates and medium corresponded to the same amount of culture. Coomassie-stained gels were imaged using a GS800 calibrated densitometer (Biorad). Visualisation of immunoblot signals was carried out using a ChemiDoc XRS+ system. Images were processed and densitometry was performed using Quantity One software (Biorad).

### Urea extraction

Urea extraction assays of isolated membrane fractions were performed as described in [19]. Cells were grown, induced for recombinant protein expression and collected by low speed centrifugation as described above. Pellets were resuspended in ice-cold TE buffer (10 mM Tris-HCl, pH 8.0, 1 mM EDTA) and suspensions were sonicated on ice using a tip sonicator (Branson 250 sonifier). The resulting lysates were first subjected to low-speed centrifugation (1,500 x g) at 4°C for 10 min to remove unbroken cells and debris, after which the supernatant was subjected to high-speed centrifugation (130,000 x g) at 4°C for 20 min. The pelleted membrane material was resuspended in ice-cold TE buffer to yield the isolated cell envelope fraction. Extraction was performed by adding to a sample an equal volume of 8 M urea, 2 M glycine in 10 mM Tris PH7.6 8 resulting in a final 4 M urea concentration. Control samples were mixed with the same buffer lacking the urea. Samples were subsequently incubated for 30 min on ice and then subjected to high-speed centrifugation at 4 °C for 30 min. Pellets were resuspended in the same volume of TE and then mixed with two-times concentrated sample buffer. Samples of untreated cell envelopes, urea extracted pellets and the resulting supernatants were analysed by SDS-PAGE followed by Western blotting.

### Heat modifiability assay

Heat modifiability assays were performed as described [13]. To half of a sample of isolated cell envelopes (see above) an equal volume of two-times concentrated SDS-sample buffer was added and the mixture was heated at 96°C for 10 min (denatured sample). The other half was mixed with an equal volume of two-times concentrated sample buffer containing 0.4% SDS and kept at room temperature (native sample). Samples were separated by electrophoresis using cooled PAGE gels containing no SDS in the acrylamide gels and normal SDS concentration in the running buffer.

### Proteinase K treatment

Proteinase K treatment of whole cells was performed as described in [12]. Cells induced for protein production were collected by low speed centrifugation and resuspended in ice-cold digestion buffer (50 mM Tris-HCl, pH 7.5, 100 mM NaCl). Proteinase K (100 μg/ml) was added to a part of the sample, which was then incubated on ice for 60 min or at 37 °C for 30 min, whereas a control sample was mock-treated with digestion buffer and kept on ice. The reactions were stopped by addition of PMSF (0.1 mM). Samples were then incubated on ice for another 10 min, after which they were TCA-precipitated and analyzed by SDS-PAGE and Coomassie staining.

### Immunofluorescence microscopy

One mL of an induced culture was collected by centrifugation, fixed using fomaldehyde and incubated for analysis using immunofluorescence microscopy with α-Myc antiserum as described previously [16]. As secondary antiserum served goat anti-rabbit antiserum conjugated to Alexa488 was used. Images were recorded on a Olympus BH-2 microscope.

## Results and discussion

### β-domain fusions used in this study

The five selected autotransporters for our comparative studies came from various backgrounds, but all have been used previously for the secretion of recombinant protein sequences [23, 25, 31–37]. We fused three model passenger domains to the β-domains of Hbp (Hbpβ), Ag43 (Ag43β), IgaP (IgAPβ), EstA (EstAβ) and Hia (Hiaβ) at fusion points that were defined in studies by others using those β-domains for export of heterologous proteins. For IgAP we fused passengers upstream of position 1124 (all positions mentioned refer to positions in the full-length amino acid sequences) yielding IgAPβ1124) (Fig 1). It starts 2 amino acids (AA) downstream of the autocatalytic cleavage site observed for IgAP [43]. Importantly, this position is used in many studies that use IgAPβ for export of heterologous proteins (*e*.*g* [32, 44]). For Ag43β, Ramesh et al [36] selected the C-terminal 700–1039 residues of the full length Ag43 protein (Ag43β(700)). This region includes a predicted β-barrel and α-helical segment as well as a linker sequence of 10 amino acids upstream of the predicted α-helix. Furthermore, for both IgAPβ and Ag43β we included a shorter variant of the β-domain encompassing the β-core (*i*.*e*. the 12-stranded β-barrel preceded by an α-helical segment; see the Introduction). For both proteins a crystal structure was not available, but the β-core domain of IgA protease, IgAPβ(1245), was described to start at amino acid 1245 based upon protease-digestion analysis [4] and can be found in the outer membrane of some strains of *Neisseria meningitidis* expressing IgAP [8]. This position has also been used as a fusion point for heterologous passengers before [34]. The start at position 710 for the predicted Ag43 β-core, Ag43β(710) was based upon published secondary structure predictions [36]. We confirmed secondary structure predictions for both IgAPβ(1245) and Ag43β(710) using the program PsiPred [45].

The EstA homologue used for export of heterologous proteins derived from *Pseudomonas stutzeri* A15 [35], but we opted to use the EstA of *P*. *aeruginosa*, because its crystal structure has been solved [7]. Based upon the study by Nicolay, the EstAβ constructs contained residues 314–646 of EstA, which are predicted to include a β-barrel preceded by a 52-AA long α-helical segment. As a fusion point for the Hbp β-domain constructs (Hbpβ) we used Ser^1091^ of Hbp. This position is in an α-helical segment and located 10 amino acid residues upstream of the autocatalytic cleavage site that separates the passenger from the β-domain (Fig 1), but downstream and outside of the β-helical stem region of the passenger [22].

Not many published studies address the use of trimeric autotransporter β-domains to export heterologous sequences to the cell surface. Trimeric autotransporter Hia was selected because its β-domain (Hiaβ) has been shown to support the export of the passenger domain of monomeric autotransporter Hap of *H*. *influenzae* to the cell surface [33]. Other trimeric β-domains facilitated export of non-cognate trimeric autotransporter passenger domains [46, 47]. We, therefore, included in our studies two versions of the C-terminal domain of Hia; Hiaβ and Hiaβ(3×). The Hiaβ constructs used the 101-AA long C-terminal part of Hia starting at Ile^920^. According to the crystal structure [15] this segment comprises an α-helix followed by four β-strands that trimerizes to form a 12-stranded β-barrel. The lumen of the β-barrel is occupied by three unstructured loops that are connected to three α-helices (S1 Fig). These three loops could potentially hinder secretion of heterologous fusion partners. To prevent such steric hindrance, we designed a monomeric Hiaα derivative, called Hiaβ(3×) (S1 Fig). Its design was inspired by the observation that duplication of 8-stranded OmpX resulted in a functional 16-stranded β-barrel [48]. Hiaβ(3×) includes a single α-helix (Ala929-Gln958 of Hia) and three translationally fused repeats of the four Hiaβ β-strands linked via a short 4-AA linker (GSPG). In theory, the Hiaβ(3×) constructs would fold into a monomeric 12-stranded barrel to accommodate a single heterologous passenger domain fused to one loop and α-helix.

All β-domain DNA constructs contained a 5’-*Spe*I restriction site for fusion to the different passenger domain-encoding sequences and all were cloned into the same plasmid background (pEH3) under control of an IPTG-inducible *lac* promoter and downstream of a sequence encoding the endogenous Hbp signal sequence for translocation across the inner membrane (See Fig 1 for an overview of the constructs used in this study).

### Expression and folding of Myc-tag β-domain fusions

**Fig 2:**
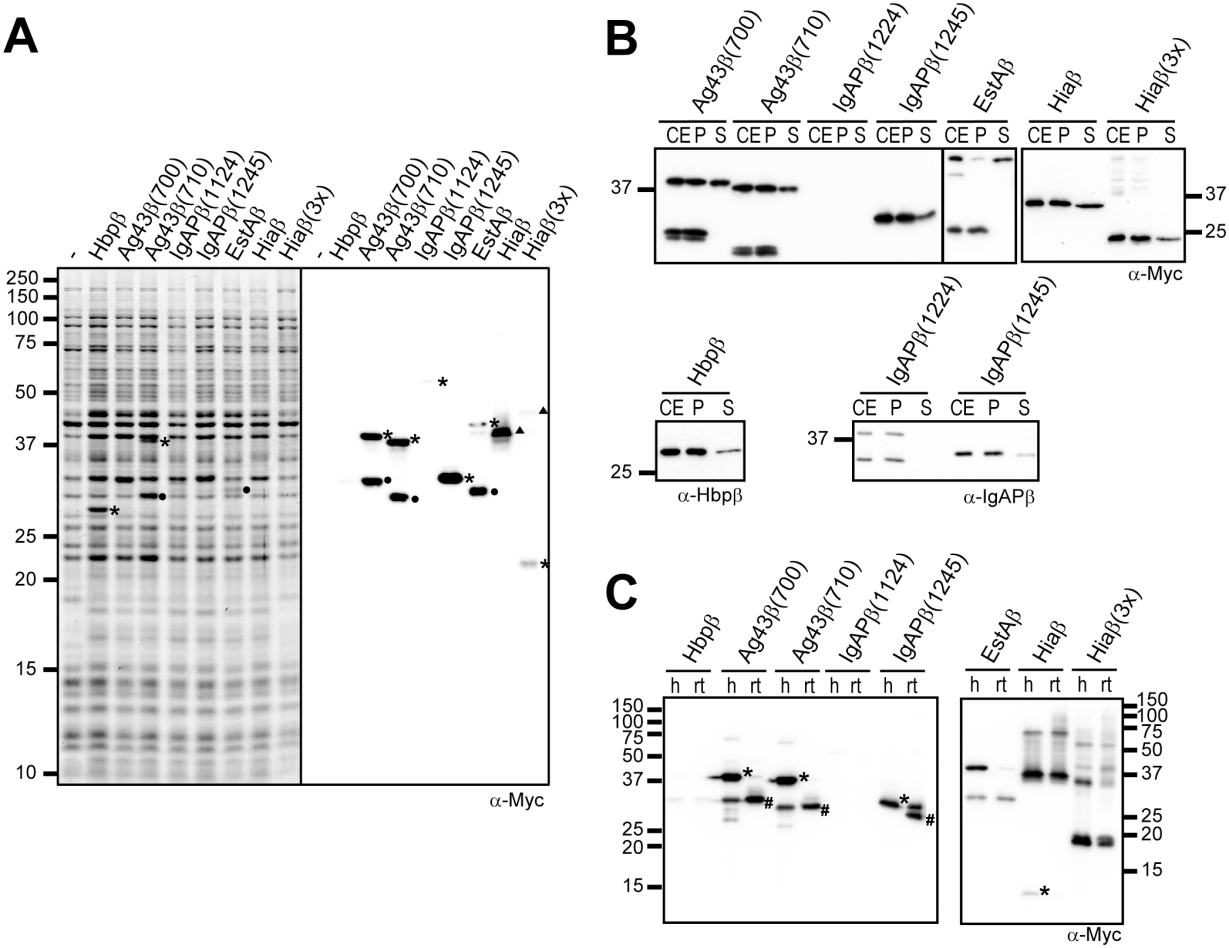
Expression, outer-membrane insertion and folding of Myc-β domain fusions. **(A)** Coomassie-stained SDS-PAGE gel (left panel) and Western blot incubated with antiserum against the myc tag (α-Myc; right panel) to detect expression of Myc-β-domain fusions in MC1061 cells. Indicated are Myc-β-domain fusions (*), degradation products (●) and bands of higher apparent molecular wheight (▲). **(B)** Western blots incubated with antisera against the myc tag (α-Myc), the Hbpβ (α-Hbpβ) and IgAPβ (α-IgAPβ) to assess insertion into the outer membrane. CE, isolated cell envelopes; P, pellet fractions of cell envelopes after Urea extraction; S, supernatant fractions of cell envelopes after Urea extraction. **(C)** Western blots incubated with α-Myc to assess heat-modifiability of membrane-inserted Myc-β-domain fusions. Samples of isolated cell envelopes were either heated at 98 °C (h) or kept at room temperature (rt). The positions of bands representing denatured proteins (*) running at the position expected from the calculated molecular wheight and folded proteins (#) running at a lower position are indicated.

We first determined whether the eight β-domain constructs were expressed and targeted to the outer membrane when fused to the short and structurally simple Myc tag of ~1.8 kDa. Similar constructs have been shown to result in efficient export of the tag to the cell surface [34]. A DNA construct was made encoding an 18-AA peptide in between the Hbp signal sequence and the various β-domain constructs that included the 10-AA Myc epitope flanked by glycine and serine residues (Fig 1). The resulting plasmids were introduced in *E*. *coli* K12 strain MC1061 and expression was induced by adding IPTG. Whole cell lysates were analysed by SDS-PAGE followed by Coomassie staining or western blotting (Fig 2). Expression of the Myc-tag constructs yielded detectable bands on a Coomassie-stained gel for constructs Myc-Hbpβ, Myc-Ag43β(710), Myc-IgAPβ(1245), and Myc-EstAβ, which suggested reasonable expression levels (Fig 2). The apparent molecular weight of the detected bands matched to the calculated molecular weights of the Myc-Ag43β(710) and Myc-IgAPβ(1245) fusions without signal peptide (Table 1). For Myc-Ag43β(710) and Myc-EstAβ a lower running band was detected at ~32 and ~33 kDa, respectively, suggesting proteolytic processing or degradation. The EstAβ product included the Myc-tag (see below). The running of Hbpβ at ~31 kDa, a position indicating the absence of the Myc-tag passenger (Table 1), is explained by the natural autocatalytic cleavage within Hbpβ [22] resulting in the release of the Myc tag. A similar intra-barrel cleavage has been observed for other short Hbpβ constructs [21, 23]. No Coomassie-stained bands were detected for IgAPβ(1124), Ag43β(700), Hiaβ en Hiaβ(3×), indicating low expression levels or degradation.

To further address expression, western blot were prepared and incubated with α-Myc antiserum. The blots revealed bands for all constructs except Myc-Hbpβ, albeit very weakly for IgAPβ(1124) (Fig 2). No band for the Myc-Hbpβ construct was detected, as expected from a release via autocatalytic cleavage. Indeed, probing a blot with α-Hbpβantiserum resulted in the detection of a single Hbpβ band at ~31 kDa (Fig 2). Both Myc-Ag43β constructs were detected in the the western blot, which showed equally strong bands at the expected positions of ~37 and ~38 kDa (Table 1), and distinct bands at ~32 and ~30 kDa, which presumably result from proteolytic degradation. The Ag43β(700) bands were likely not noticed on the Coommassie-stained gel, because they co-localized with other highly expressed proteins. The Myc-IgAPβ(1124) was only detected very weakly on blot at ~53 kDa, indicative of proteolytic degradation. Its position on gel was higher than expected from its calculated molecular weight (Table 1), but this has been observed more often for IgAPβ-domains [30,35]. Expression of Myc-IgAPβ(1245) resulted in a clear band on gel and blot at its expected position of 33 kDa, which corroborates with observations in literature of OmpT-mediated degradation of IgAPβ(1124) and not of IgAPβ(1245) [4]. The ~39-kDa Myc-EstAβ construct could only weakly be observed on blot, whereas a prominent band was detected at ~33kDa on both blot and Coomassie-stained gel, indicative of proteolytic degradation. Finally, the Myc-Hiaβ construct yielded a band at ~38 kDa on the blot, which could be a trimer of the ~12-kDa protein. Previously, trimeric complexes of full-length Hia have been detected on blots despite boiling of samples in the presence of SDS [33]. Furthermore, Myc-Hiaβ(3×) ran at its expected molecular weight of ~23 kDa as well as higher bands, suggesting multimers or aggregates to be formed. However, detection was only weak suggesting either low expression or degradation.

The appearance of degradation products for and weak expression of some of the constructs could indicate that these proteins did not fully insert into the outer membrane, making them targets for degradation by periplasmic and outer membrane-based proteases like DegP and OmpT. Our previous work has shown that Hbp mutants that are blocked in outer membrane secretion are degraded by the periplasmic protease/chaperone DegP [16, 19]. As part of the cell-envelope stress response, DegP is involved in quality control and degradation of unfolded outer membrane proteins prior to their membrane insertion [40]. Furthermore, heterologous autotransporters expressed in *E*. *coli* have been shown to be cleaved by outer membrane protease OmpT [4, 49]. To assess stable insertion of the Myc-β domain constructs into the outer membrane, we isolated cell envelopes and performed urea-extraction and heat-modifiability assays. Adding urea to isolated cell envelopes denatures proteins in the sample to such an extent that proteins that are not fully inserted into a membrane are extracted [12, 19]. After incubation of isolated cell envelopes in 4 M urea, we ultracentrifuged the samples to separate soluble and membrane-associated proteins and analysed the results by western blotting using α-Myc or α-Hbpβ antisera (Fig 2). We detected Hbpβ at ~31 kDa in the isolated cell envelopes, as expected, and the protein recovered primarily in the pellet fraction after urea incubation, indicative of its stable insertion into the OM (Fig 2B, bottom left panel). Except Myc-IgAPβ(1124), all other Myc-β-domain fusions were also detected in the isolated cell envelopes (Fig 2C, upper panels). Moreover, they also fractionated into the urea-extracted pellet indicating that they stably inserted into the outer membrane. A notable exception was the ~39-kDa Myc-EstAβ fusion, which clearly fractionated into the supernatant upon addition of urea. Remarkably, the ~33-kDa Myc-EstAβdegradation product was not extracted and probably represents a stably inserted protein species. Similar to what was observed on blots of whole cell samples, the Myc-Hiaβ construct appeared to run as a trimer, which appears very stable, since it is not resistant to 4M Urea. Since we could not detect IgAPβ(1124)-derived bands using the α-Myc antiserum, we incubated a blot containing urea-treated cell envelopes of the two IgAPβ constructs with α-IgAPβ antiserum. It revealed IgAPβ(1124) protein species running at ~40 and ~30 kDa that lack the Myc tag. The ~30 –kDa species could represent the IgAPβ-core, since its size is very similar to the Myc-IgAPβ(1245) construct (Fig 2C, panel bottom left). Importantly, the IgAPβ protein species all were present in the urea-extracted pellet, which suggests stable insertion into the outer membrane.

The heat-modifiability assay is based upon the observation that outer membrane β-barrel proteins maintain their structure when incubated in a buffer with a low concentration of SDS (0.4%) at room temperature. Consequently they run at a different, usually lower, position during PAGE when compared to heat-denatured samples [50]. The heat-modifiability of several β-domains of monomeric autotransporters has been established experimentally, among which Hbpβ [5, 6, 12, 21, 50, 51]. To assess β-barrel formation by the Myc-β-domain fusions, we compared boiled and non-boiled cell-envelope samples of cells expressing these constructs. These were run on semi-native SDS-PAGE gels that are prepared with reduced amounts of SDS and heat-modifiability was detected by western blotting using α-Myc antiserum. Consistent with our previous results (see above) the Myc-Hbpβ and Myc-IgAPβ(1124) constructs were not detected. The β-domains of the other monomeric autotransporters showed heat-modifiability (Fig 2D, left panel), with a faster running band detected in the non-boiled samples. Furthermore, boiling the samples in the presence of 0.4% SDS did not fully denature the proteins, which underscores the stability of the β-barrels formed. Remarkably, the ~39 kDa band detected in the heat-denatured EstAβ-Myc sample was absent in the sample that was kept at room temperature (Fig 2D, right panel), but it could not be detected at an alternative position in gel. Possibly, non-denatured EstAβ-Myc aggregated and did not enter the SDS-PAGE gel matrix, which would be in line with the observed sensitivity to urea-extraction of EstAβ-Myc. Overall, our results suggested that the ~39 kDa protein did not integrate into the outer membrane. The Hiaβ-Myc construct in the non-heated sample ran at the same position as in the heated sample (Fig 2C, right panel), but in the heated sample a band was detected at ~12 kDa likely representing some monomeric Hiaβ-Myc. Similarly, no change of position was observed for the ~21 kDa monomeric band in the non-heated versus the heated Myc-Hiaβ(3×) samples. Heat-modifiability for trimeric autotransporter β-domains has not been tested before, amongst others because these are not proteolytically separated from their passengers and remain as full-length proteins in the outer membrane. Yet, their resistance to urea extraction observed here suggests that they are inserted in the membrane.

Overall the results suggest that the β-domain constructs, with the exception of EstAβ were inserted into the outer membrane in a folded conformation, although the latter is difficult to assess for the Hia constructs. Furthermore, only the Myc-Hbpβ, -Ag43β and -IgAPβ(1245) reached reasonably high expression levels and outer-membrane insertion, whereas expression and insertion for the other constructs is limited.

### Expression of the Hbp passenger fused to different β-domains

To establish whether the different β-domain constructs can secrete larger cargo than the rather small Myc-tag, we fused them to the Hbp passenger (Fig 1). Previously, we had fused it to the β-domain of monomeric *E*. *coli* autotransporter EspP (EspPβ), which like Hbp belongs to the SPATE family of monomeric autotranporters [12]. The EspPβ could both secrete and process the Hbp passenger, although expression levels of Hbp-EspPβ were slightly reduced compared to wild-type Hbp. Along a similar line, fusing the passenger of monomeric autotransporter Hap to the Hia β-domain resulted in cell-surface exposure of the passenger on both *E*. *coli* and *H*. *influenza* cells as shown by FACS analysis and Hap-mediated binding to cultured human epithelial cell lines [34]. Hap is not a SPATE, but its passenger closely resembles the passengers of that protein family. Together, these results suggested that an autotransporter passenger may be secreted using a non-cognate β-domain and we, therefore, compared the secretion of the Hbp passenger fused to its cognate β-domain and to the other β-domain constructs.

**Fig 3:**
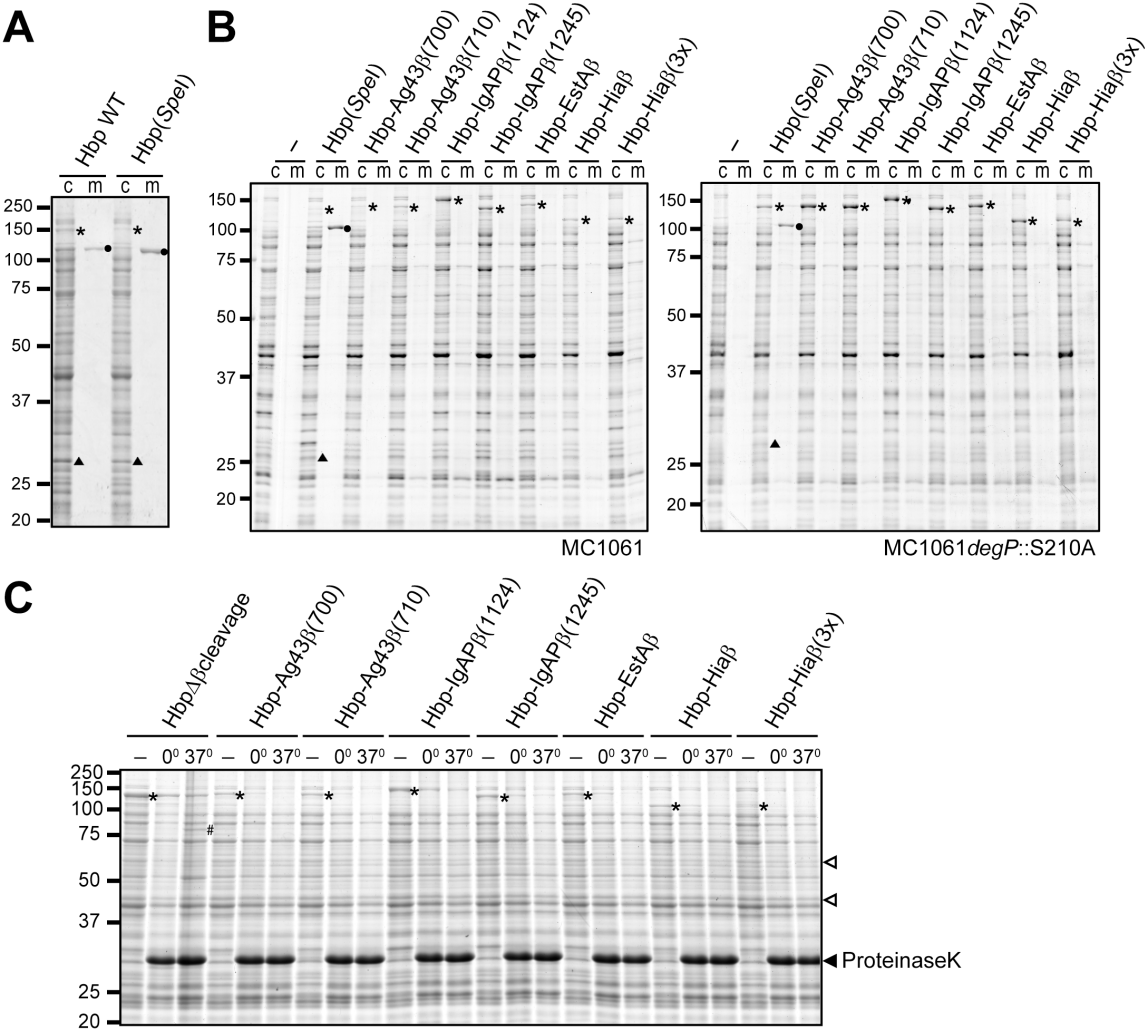
Expression and surface-accessibility of Hbp-β domain fusions. **(A)** Coomassie-stained SDS-PAGE of whole cell lysates (c) and culture supernatants (m) of MC1061 cells expressing wild-type Hbp and Hbp(*Spe*I). **(B)** Coomassie-stained gel of whole cell lysates (c) and culture supernatants (m) of MC1061 (left panel) or MC1061 *degP*::S210A (right panel) expressing Hbp-β-domain fusions. **(C)** Coomassie-stained gel of whole cell lysates of cells of MC1061 expressing the Hbp-β-domain fusions incubated with proteinase K, either for 60 min on ice (0°) or 30 min at 37 °C (37°). Included are also untreated controls (-).The positions of bands representing unprocessed Hbp passenger-β-domain fusions (*), and processed Hbp passenger (●) and Hbpβ (▲) are indicated. The latter are only detected for Hbp(*Spe*I). In panel C, the prominent ~79-kDa of Hbp-Δβcleavage degradation product is indicated by (#), the position of the proteinase K bands is indicated by the closed arrowhead, whereas the open arrowheads indicate the control bands used for densitometric analysis.

To enable fusion of the Hbp passenger to the different β-domain constructs, we engineered a *SpeI*-fusion site in the *hbp* sequence (Fig 1) at the position encoding Ser^1091^, which is downstream of the β-helical stem structure formed by the passenger domain and 10 residues upstream of the autocatalytic cleavage site that separates the Hbp passenger and β-domain [22]. As a result of the cloning procedure, the encoded Hbp variant (Hbp(*SpeI*)) carries a substitution of Ile^1090^ for Thr. This substitution did not affect the expression and processing of Hbp, given the appearance of similar levels of the cleaved β-domain in cell samples (Fig 3). However, the mutation did affect the level of processed Hbp passenger associated with the cell surface [12, 16], since less of the processed passenger was retained in the cell fraction compared to wild-type Hbp. This effect has been observed for other Hbp mutants as well [12] and likely reflects a change in the non-covalent interactions that keep the processed passenger bound at the cell surface. Subsequently, the *Spe*I-fusion site was used to engineer fusions of between the Hbp passenger and the other β-domain constructs, yielding Hbp-Ag43β(700), Hbp-Ag43β(710), Hbp-IgAPβ(1124), Hbp-IgAPβ(1245), Hbp-EstAβ, Hbp-Hiaβ and Hbp-Hiaβ(3×), respectively (Fig 1).

IPTG-induced expression of the fused Hbp-β-domain constructs in MC1061 did not affect the growth of the bacterial cultures, except for cells expressing Hbp-IgAPβ(1245) and Hbp-Hiaβ(3×) (S2 Fig). The latter cultures reached optical densities of OD_600_ ~1.5 after IPTG induction at OD600 ~0.3, where other cultures reached values of 2.5–3.0 after induction. Apparently, expression of the latter fusion proteins interfered with optimal cell growth. Production of the fusion proteins was analyzed by SDS-PAGE and Coomassie staining (Fig 3). With the exception of Hbp(*Spe*I), autocatalytic cleavage of the constructs was not expected to occur, because either the required protease domain (IgAPβ or the targeted cleavage site (Ag43β) is missing in the construct or the original autotransporter is not proteolytically processed at all (EstAβ and Hiaβ. Consistently, expression of these fusions did not result in detection of released Hbp passenger in the culture supernatant samples, while clear bands were detected in the cell samples (Fig 3) at positions corresponding to the calculated molecular weight of the passenger-β-domain fusions (without signal peptide; Table 1). Highest expression levels were observed for Hbp-IgAPβ(1124) and -IgAPβ(1245) constructs. An exception was Hiaβ(3×)-Hbp, for which we observed a ~120 kDa band, that is possibly a degradation product with a size that is similar to what expected for Hbp-Hiaβ (Table 1).

The different expression levels detected could result from difficulties in outer membrane insertion and subsequent periplasmic degradation by DegP, as has been shown for Hbp [16, 19]. To test this, we expressed the constructs in MC1061 *degP*::*S210A*, which expresses a mutated version of DegP, which lacks its protease activity through mutation of the active site but retains its activity as a chaperone [40]. Expression of the constructs in MC1061 *degP*::*S210A* affected growth of cultures more than expression in wild-type MC1061, most likely due to periplasmic accumulation of non-secreted proteins that stalled at the outer membrane, as previously suggested for wild-type and mutant Hbp proteins [16, 19]. Samples of MC1061 *degP*::*S210A* expressing Hbp(*Spe*I), indeed, showed slightly reduced levels of secreted Hbp passenger and processed Hbpβ on Coomassie-stained SDS-PAGE gels when compared to expression in wild type MC1061, while levels of unprocessed Hbp increased (Fig 3). Overall, the levels of Hbp-IgAPβ(1124) did not change much in MC1061 *degP*::*S210A* mutant background, suggesting that its biogenesis was quite efficient. In contrast, all other Hbp-β-domain constructs showed a markedly increased expression level in MC1061 *degP*::*S201A* (Fig 3, compare panels B and C), which suggested that the low expression levels in wild type MC1061 were the result of DegP-mediated degradation of periplasmic secretion-incompetent intermediates. Furthermore, Hiaβ(3×)-Hbp in the *degP* mutant strain again degraded into a ~120-kDa shorter form, indicating that DegP is not involved in this degradation.

The detection of the fusions in wild type MC1061 suggested that all constructs exported the Hbp passenger to the cell surface at least to some extent. We performed a proteinase K accessibility assay to monitor such exposure. In this assay, proteinase K is added to a suspension of intact cells in order to degrade proteins that are exposed on the bacterial cell surface, including properly exposed Hbp-β-domain fusions. Previously, we have used this assay to assess surface exposure of Hbp mutants and showed that externally added proteinase K degrades exposed Hbp passenger at the cell surface, while non-exposed Hbp mutants that accumulated inside the cell were not degraded [19]. Here, we incubated cultured MC1061 cells expressing the Hbp-β-domain fusions with 100 μg/ml proteinase K either for 60 min on ice, or for 30 min at 37°C, after which whole cell lysates were analysed on Coomassie-stained gels (Fig 3C). As a control for surface exposure of the Hbp passenger served the Hbp-Δβcleavage mutant [12, 16, 19]. This Hbp mutant has a mutation in the autocatalytic cleavage site, resulting in a non-processed passenger, like expected for the other β-domain fusions to the Hbp passenger. Proteinase K degraded a large part of the exposed Hbp-Δβcleavage protein, indicative of its accessibility at the cell surface (Fig 3). The 30-min 37°C sample further showed a specific ~79-kDa degradation product seen earlier [19] that was not present in the sample incubated for 60 min on ice (Fig 3). Similar to Hbp-Δβcleavage, the bands corresponding to the other Hbp-β-domain fusions decreased in the presence of proteinase K suggesting that they were at least in part exposed on the cell surface. The overall protein pattern in the Coomassie-stained gels appeared not affected by proteinase K, suggesting that in presence of proteinase K most cells remained intact. This was corroborated by densitometric analysis of two non-related protein bands in the Coomassie-stained gels that showed different intensities and appeared not affected by the presence of proteinase K (Fig 3). The density measured for these two bands did not differ more than 10% to the mean density detected. In a control experiment we further assessed degradation of the periplasmic chaperone SurA and outer membrane-based protein OmpA on western blots. Periplasmic domains of OmpA become sensitive to Proteinase K when cells lose their integrity [23]. We tested cells expressing Hbp-Δβcleavage or Hbp-IgAPβ(1245), the latter because growth of cells expressing this constructs was most affected (S2 Fig). Densitometric analysis of the blots indicated that OmpA levels remained unaffected, whereas SurA was affected slightly with levels decreasing to ~80% of untreated cells when proteinase K was present. In comparison, proteinase K degraded the β-domain fusions detected on the Coomassie-stained gels always to levels lower than 80% of untreated samples, albeit to different extents.

Overall, the results indicated that most non-cognate β-domains were able to support secretion of the Hbp passenger to some degree, with the IgAPβ(1124) construct being the most efficient. The secreted IgA protease passenger itself is a SPATE-like serine protease with a structure very similar to Hbp [52] and its β-domain may, therefore, be suited for a SPATE passenger. Furthermore, the differences in expression levels of the two IgAPβ constructs and, to a lesser extent, of the two Ag43β constructs indicated that it is important to select an optimal fusion point, since each variant shows different results.

### Expression of V_HH_ domains as heterologous passenger

**Fig 4:**
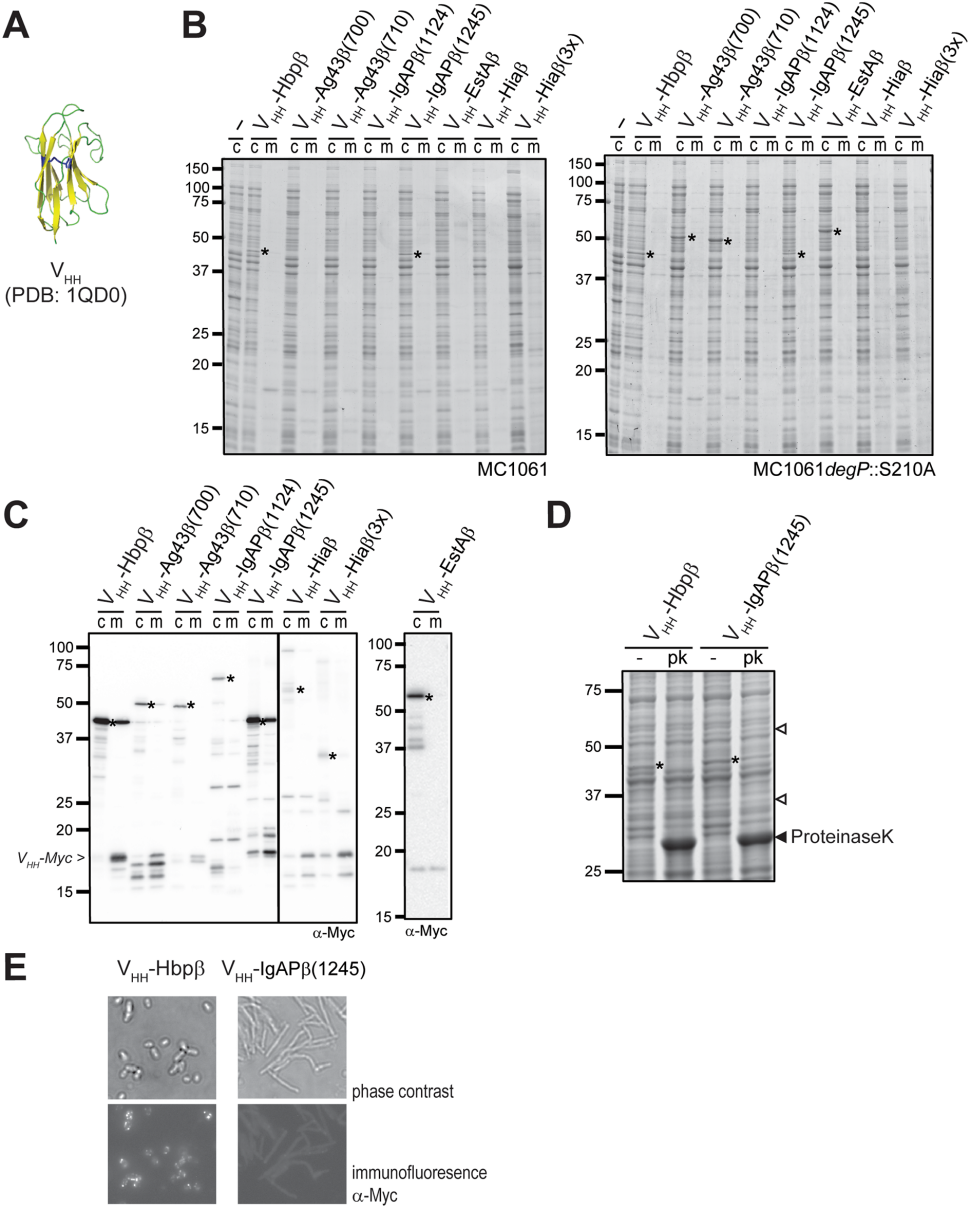
Expression and surface-accessibility of V_HH_-β domain fusions. **(A)** Cartoon of the structure of the V_HH_ domain fused to the eight β-domain constructs. The disulfide bond that stabilizes the secondary structure and is likely formed in the periplasm is highlighted in blue. **(B)** Coomassie-stained SDS-PAGE of whole cell lysates (c) and culture supernatants (m) of MC1061 (left panel) or MC1061 *degP*::S210A (right panel) cells expressing V_HH_-β-domain fusions. Indicated are the detectable V_HH_-β-domain fusions (*). **(C)** Western blots incubated with α-Myc of whole cell lysates (c) and culture supernatants (m) of MC1061 cells expressing the V_HH_-β-domain fusions (*). The expected position of a processed V_HH_-Myc fusion of 18 kDa is indicated on the left. Note that the right panel is derived from a different blot. Compare the results also with Fig 2S that shows that blots of constructs expressed in *E*. *coli* strains DHB4 and DHBA yielded a very similar detection pattern. **(D)** Coomassie-stained SDS-PAGE gels of whole cell lysates of cells of MC1061 cells expressing the V_HH_-Hbpβ or V_HH_-IgAPβ(1245) (*) incubated with 100 μg/ml proteinase K (pk) or with buffer (-) for 30 min at 37 °C. The position of the proteinase K bands is indicated by the closed arrowhead, whereas the open arrowheads indicate the control bands used for densitometric analysis (**E)** Microscopic images of MC1061 cells expressing V_HH_-Hbpβ (right panels) or V_HH_-IgAPβ(1245) (left panels). The top panels are phase-contrast images, the lower panels are immunofluorescent images. Contours of cells are visible due to background autofluoresence.

Autotransporters are considered attractive systems for production and export of relevant recombinant proteins [1, 2]. To test the eight β-domain constructs for their ability to secrete a recombinant protein of biotechnical interest that has to be fully folded to be functional, we constructed fusions to a nanobody or V_HH_ domain [38]. The crystal structure of this V_HH_ domain (PDB 1QD0) revealed an Ig-like domain of ~19 by ~35 Å that includes two cysteine residues that form a disulfide bond that stabilizes the structure [53] (Fig 4). Of note, in Gram-negative bacteria disulfide bonds are formed in the periplasm, so prior to the translocation of the autotransporter passenger across the outer membrane. Furthermore, our previous research showed that insertions in Hbp of fusion partners with a propensity to form stable tertiary structures, such as the V_HH_ domain, led to degradation of the passenger in the periplasm. For example, inclusion of a hairpin of two α-helices stabilized by a disulfide bond blocked secretion of the Hbp passenger [17], as did inclusion of a tightly-folded calmodulin domain [16]. Insertion of stably folded protein segments in other autotransporters also blocked the secretion process [29, 54]. On the other hand, fusions of V_HH_ domains and other single-chain antibody fragments directly to β-domains including Ag43β(700), IgAPβ(1124) and IgAPβ(1245) were reported to be surface exposed, despite the presence of disulfide bonds [30, 32, 34, 36, 37, 44, 55]. These conflicting results prompted us to systematically compare the secretion of a V_HH_ domain when fused directly to β-domain constructs.

We fused a V_HH_ and a Myc tag for detection to the eight β-domain constructs and tested expression in *E*. *coli* MC1061 (Fig 1). The induction of expression of the constructs only slightly affected growth of cultures when compared to the Hbp-fusion constructs, with OD_600_ values ranging from 1.5 to 2.5 (S3 Fig) suggesting a comparable impact of the expression of the V_HH_ fusions on cell viability, again with the exception of the culture expressing the V_HH_-Hiaβ(3×) construct, which showed a more severely reduced growth. Expression was subsequently analysed on Coomassie-stained gels. The gels showed unprocessed V_HH_-Hbpβ and V_HH_-IgAPβ(1245) fusions at positions that matched their calculated molecular weights (Table 1) while the other fusions were not detected indicative of degradation (Fig 4). To test periplasmic degradation by DegP, we expressed the Vhh fusions in MC1061 *degP*::*S210A* and analysed whole cell lysates on Coomassie-stained gels (Fig 4). The expression levels of the V_HH_-Hbpβ and V_HH_-IgAPβ(1245) constructs appeared similar to wild-type MC1061. Furthermore, clear protein bands could now be detected for the two V_HH_-Ag43β and the V_HH_-EstAβ fusions confirming their periplasmic degradation by DegP in wild-type MC1061. Western blots incubated with an anti-Myc antiserum revealed protein bands at the expected positions for unprocessed V_HH_-β-domain proteins for all fusions (Fig 4), but with lower levels of detection for the fusions not detected on Coomassie-stained gels. Furthermore, we observed various degradation products, as well as the presence of cellular proteins in the culture medium samples, probably due to cell lysis. Unexpectedly, no autocatalytic processing of the V_HH_-Hbpβ fusion into separate V_HH_ and Hbpβ domains was observed on the Coommassie-stained gels, despite the autocatalytic cleavage observed for other Hbpβ fusions. On blot we did detect a ~18-kDa band that could represent the secreted V_HH_-Myc passenger, since it appeared in the culture supernatant and not in the cell samples. Similar putatively secreted bands of the size of the V_HH_ domain were specifically detected in the culture supernatants of cells expressing V_HH_- Ag43β(700), V_HH-_Ag43β(710) and V_HH_-IgAPβ(1245). In view of the absence of a natural processing site in these constructs, such a processing could have resulted from an external protease like OmpT. Overall, the results suggested that V_HH_-Hbpβ and V_HH_-IgAPβ(1245) were exposed at the cell surface at least to some degree, whereas the two Ag43β-V_HH_ fusions may be exposed too dome, but clearly lower extent.

The observed poor expression of most of the fusions could be due to formation of the disulphide bond in the V_HH_ domain and subsequent blocking of the translocation across the outer membrane. To test this assumption, the constructs were analyzed in the DsbA knockout strain DHBA and its isogenic wild-type DHB4 (S3 Fig). Similar to what was found for MC1061, most of the V_HH_-β-domain fusions were not detected in these strains on Coomassie-stained gels except for V_HH_-Hbpβ and V_HH_-IgAPβ(1245). However, they could be detected on a blot probed with α-Myc, which yielded a very similar picture to that of MC1061 (S3 Fig). Interestingly, the absence of DsbA resulted in better detection of the two V_HH_-Ag43β constructs suggesting an improved assembly into the outer membrane, but not to levels detectable on Coomassie-stained gels. The expression of the other constructs did not change in absence of DsbA. We concluded, therefore, that disulfide bond formation is not a major cause for the poor expression of these constructs. Rather, these β-domains seem less well suited for the secretion of the V_HH_ domain.

Both V_HH_-Hbpβ and V_HH_-IgAPβ(1245) were expressed at Coomassie-detectable levels, which could indicate stable expression and display of V_HH_ at the cell surface. However, an alternative explanation for the detection of non-processed V_HH_-Hbpβ fusion could be that translocation across the outer membrane was blocked, since this step precedes and is a prerequisite for the subsequent processing of the wild-type Hbp passenger [19, 54] and this was not observed. To assess the cell surface exposure of both fusions we performed a proteinase K accessibility assay as well as immunofluorescence microscopy to detect the Myc tag on the surface of MC1061 cells expressing the V_HH_-Hbpβ and V_HH_- IgAPβ(1245) constructs. Externally added proteinase K almost fully degraded the Hbpβ-V_HH_ fusion to ~15% of the non-treated sample, as indicated by densitometric analysis, indicating efficient cell surface exposure (Fig 4). In contrast, most of the V_HH_-IgAPβ(1245) remained detectable and densitometric analysis indicated a decrease of ~45% of the untreated cells. Control bands on the Coomassie-stained gels showed no degradation for the cells expressing the V_HH_-Hbpβ fusion, and degradation to 75% for the cells expressing the V_HH_-IgAPβ(1245) fusion. We further assessed the degradation of OmpA and SurA on blot (S3 Fig). Densitometric analyses indicated that OmpA was slightly affected at 90% of untreated cells for both fusions, whereas the SurA levels decreased to ~55% of untreated cells, suggesting that the V_HH_-β-domain fusions affected stability of the cells to a greater extent than the Hbp-β-domain fusions did. Nevertheless, the degradation measured for the V_HH_-Hbpβ was much greater than the controls, suggesting that the V_HH_ domain was exposed at the cell surface. We then used immunofluorescence microscopy to further investigate the surface exposure of the V_HH_-domains of V_HH_-Hbpβ and V_HH_-IgAPβ(1245) by in incubating MC1061 cells expressing V_HH_-Hbpβ and V_HH_-IgAPβ(1245) with α-Myc antiserum and a secondary antibody conjugated to the fluorescent dye Alexa488 (Fig 4). Surprisingly, the phase-contrast images showed elongated MC1061 cells when expressing the V_HH_-IgAPβ(1245) fusion, indicating physiological problems resulting from that expression. Apparently, the expression of the fusion interfered with normal cell growth, although its effect was not influencing cell viability per se as judged from the growth curves. In contrast, cells expressing the V_HH_-Hbpβ fusion (Fig 4E, top left image) or expressing Hbp-Δβcleavage looked normal [16]. Importantly, only the V_HH_-Hbpβ showed a punctuated fluorescent signal, suggesting accessibility of the Myc-tagged V_HH_ domain at the cell surface for antibody binding, while the V_HH_-IgAPβ(1245) fusion was not detected by the externally added antibodies. The Vhh-IgAPβ(1245) was clearly expressed by the cells, and this result, therefore suggests, that the produced V_HH_-IgAPβ(1245) accumulates for a large part intracellularly, corroborating the results obtained by the proteinase K experiment.

Overall, the outcome of our comparison favors the use of Hbpβ for export of V_HH_ domains, even over the use of Ag43β and IgAPβ fusions, for which successful secretion has been reported [30, 32, 34, 36, 37, 44, 55]. In these studies, expression of single-chain antibodies fused to IgAPβ(1124) [32, 44] and IgAPβ(1245) [35] were tested with immunoblots, which makes judging and comparing of expression levels difficult. Other studies of V_HH_ or single chain antibodies fused to the EhaA or BrkA β-domains [30, 34, 37] also assessed expression and cell-surface exposure on blots and not Coomassie-stained gels, making direct comparisons to the results presented here difficult. In the studies, expressing the V_HH_-IgAPβ fusion in strains lacking OmpT or DsbA improved expression [32, 34, 44], indicating that levels in wild-type strains were suboptimal. Ag43-mediated cell-surface display of a V_HH_ domain using a V_HH_-Ag43β(700) construct was confirmed by FACS analysis [36], but the results provided limited insight in the expression levels. Furthermore, the data indicated that only about half of the cells presented V_HH_ on their cell surface [36]. Another recent study showed that a 28-kDa single chain antibody fused to Ag43β remained insensitive to externally added proteases [30], which argues against the use of this domain for surface display. Surface exposure of single-chain antibodies fused to IgAPβ(1124) and IgAPβ(1245) were tested by heat-modifiability and sensitivity to externally added proteases, followed by detection of proteins on blots [32, 34, 44]. Our results, in particular regarding the differential expression levels of IgAP(1124)- and IgAPβ(1245)-constructs, corroborate the results presented in these papers, but also demonstrate that western blot detection, which is semi-quantative at best, can lead to misleading conclusions regarding the levels of expression and surface exposure. However, the additional FACS and whole-cell ELISAs in performed in those studies clearly suggested that these fusions resulted in cell surface exposure. Apparently, it matters which β-domain is chosen to fuse the V_HH_ to and secretion to the cell surface is not generally observed for all β-domains studies. The unexpected elongated cell morphology when the Vhh-IgAPβ(1245) construct was expressed has not been found in other studies. The observations could be an anomaly of using strain MC1061, or be a general result of expressing a difficult autotransporter fusion. The result suggests that microscopic analysis could be important in deciding what construct to use for efficient surface display.

In the past, we have included various recombinant proteins in the Hbp passenger, replacing the sub-domains that extend from its β-helical stem [23, 25]. Not all of the insertions tested resulted in efficient surface display of the recombinant Hbp passenger. Furthermore, in a study that compared fusions of a single-chain antibody to either the Ag43 passenger or the Ag43 β-domain the latter also was shown to result in better surface display [36]. Here we show that fusion of a V_HH_ domain directly to the Hbpβ domain results in efficient expression and clear cell-surface exposure. Furthermore, a side by side comparison of several β-domain constructs, of which some have been reported to efficiently export V_HH_-like domains, shows that Hbpβ compares well to these constructs in expression levels and surface display.

## Conclusion

In this study we compared eight autotransporter β-domain constructs of five different autotransporters for their capacity to secrete fused heterologous sequences varying in size and complexity to the cell surface. We assayed expression levels, assembly in the outer membrane and surface exposure. Our results show that the type of passenger influences the secretion capacity of a β-domain. Structure appears more important than size of the passenger fused, since the long ~110 kDa Hbp passenger, which is structurally related to the endogenous passengers of most of the β-domains used, could be exposed on the bacterial surface by most β-domains, albeit with different efficiencies. The V_HH_ domain may be much smaller than the Hbp passenger but, presumably, it attains a bulky conformation during the secretion process that could interfere with its secretion to the cell surface. Its structure is stabilized by a disulfide bond formed in the bacterial periplasm by the Dsb enzymes. Fusing this domain to the β-domains resulted in reduced expression levels for most β-domain constructs, except for Hbpβ and IgAPβ(1245). The latter two constructs showed reasonably high expression levels, but only the V_HH_ domain fused to the Hbpβ was accessible for degradation by proteinase K and externally added antibodies, indicating surface exposure. Overall, the results indicate that attaining a more bulky secondary structure could interfere with secretion and hamper cell surface display.

For Ag43 and IgAP we have tested two constructs and both configuration have been tested for fusions of heterologous proteins in earlier studies [30, 32, 34, 36, 44]. Our results suggested that for Ag43 and IgAP both β-domain configurations are compatible with cell-surface exposure of the Hbp passenger (Fig 3), but that application in the secretion of other cargo proteins could show differences in expression levels, often due to proteolytic digestion during their biogenesis (Figs 2 and 4). Apparently, the interaction of a certain passenger with the fused β-domain decides whether a fusion is successfully expressed or not. This corroborates our previous observation that the Hbp β-domain is not just an outer-membrane targeting domain [12]. Furthermore, the use of Hiaβ, derived from a trimeric autotransporter, either in its natural trimeric form or in a synthetic monomer-like configuration, resulted in detectable levels of expression and surface-exposure of the Hbp passenger, similar to what was observed with Hap-Hiaβ fusions [33]. Nevertheless, the secretion capacity and, hence, usefulness of Hiaβ to secrete recombinant passengers appeared limited.

Overall, our comparative study showed that for all passenger-β-domain fusions tested, the Hbpβ domain yielded the most consistent results. It was able to secrete all fused passengers to levels that were detectable on Coomassie-stained gels. Furthermore, the Hbpβ constructs appeared fully inserted into the outer membrane with clearly cell-surface exposed passengers. Therefore, fusion of recombinant proteins directly to the Hbpβ domain expands the Hbp toolbox for the secretion and cell surface display of heterologous protein sequences.

## Supporting information

S1 Fig Structural models and nucleotide and protein sequence of Hiaβ and Hiaβ(3×). **(A)** Structural models of the Hbpβ, Hiaβ, Hbp passenger and V_HH_ domain. The PDB codes of the depicted models are given. **(B)** Nucleotide sequence of the *SpeI-Eco*RI fragment encoding the Hiaβ construct. Restriction sites used for cloning are indicated. The encoded amino acids are given below the nucleotide sequence with the amino acids constituting the four-stranded β-sheet contributing to the barrel in red. **(C)** Nucleotide sequence of the *SpeI-Eco*RI fragment encoding the Hiaβ(3×). The encoded amino acids are given below the nucleotide sequence with the amino acids constituting the three-time repeated segment in red, blue and pink.
(PDF)

S2 Fig 2S Expression of the Hbp-β-domain fusions. **(A)** Growth curves of cultures expressing the Hbp-β-domain fusions in MC10161 (left) and MC1061 *degP*::S210A (right). Expression was induced by adding IPTG at the timepoint indicated by the dotted line. (**B)** Western blots of cell samples of MC1061 expressing HbpΔβcleavage and Hbp-IgAPβ(1245) incubated with proteinase K either for 60 min on ice (0°) or 30 min at 37 °C (37°). Included are also untreated controls (-). A blot of a single SDS-PAGE gel was cut in two parts after which the top part was incubated with α-SurA antiserumem and the bottom part with α-OmpA antiserum.
(PDF)

S3 Fig Expression of V_HH_-β-domain fusions. **(A)** Growth curves of cultures expressing the V_HH_-β-domain fusions in in MC10161 (top left), MC1061 *degP*::S210A (top right), DHB4 (bottom left) and DHBA (*dsbA*^-^; bottom right). Expression was induced by adding IPTG at the timepoint indicated by the dotted line. **(B)** Western blots of cell samples in DHB4 and DHBA (*dsbA*^-^*)* cells. Westernblots incubated with α -Myc of whole cell lysates and culture supernatants of cultures of DHB4 (left panel) and DHBA (*dsbA*^-^) (right panel) expressing the V_HH_-β-domain fusions (*) to assess their expression. **(C)** Western blot of MC1061 cells expressing V_HH_-Hbpβ and V_HH_-IgAPβ(1245) incubated with proteinase K either for 30 min at 37°C (37°). Included are also untreated controls (-). A blot of a single SDS-PAGE gel was cut in two parts after which the top part was incubated with α-SurA antiserum and the bottom part with α-OmpA antiserum. The relevant lanes were cut from a larger image to place them side by side.
(PDF)

S1 Table Primer sequences used for cloning the β-domain fusions. (PDF)
