# Supplementary material for: Comparing autotransporter β-domain configurations for their capacity to secrete heterologous proteins to the cell surface
Source: PLoS One. 2018 Feb 7;13(2):e0191622. doi: 10.1371/journal.pone.0191622 (PMC5802855; doi:10.1371/journal.pone.0191622)
Supplement: S1 Fig — (A) Structural models of the Hbpβ, Hiaβ, Hbp passenger and VHH domain. The PDB codes of the depicted models are given. (B) Nucleotide sequence of the SpeI-EcoRI fragment encoding the Hiaβ construct. Restriction sites used for cloning are indicated. The encoded amino acids are given below the nucleotide sequence with the amino acids constituting the four-stranded β-sheet contributing to the barrel in red. (C) Nucleotide sequence of the SpeI-EcoRI fragment encoding the Hiaβ(3×). The encoded amino acids are given below the nucleotide sequence with the amino acids constituting the three-time repeated segment in red, blue and pink. (PDF) [file pone.0191622.s001.pdf]

A

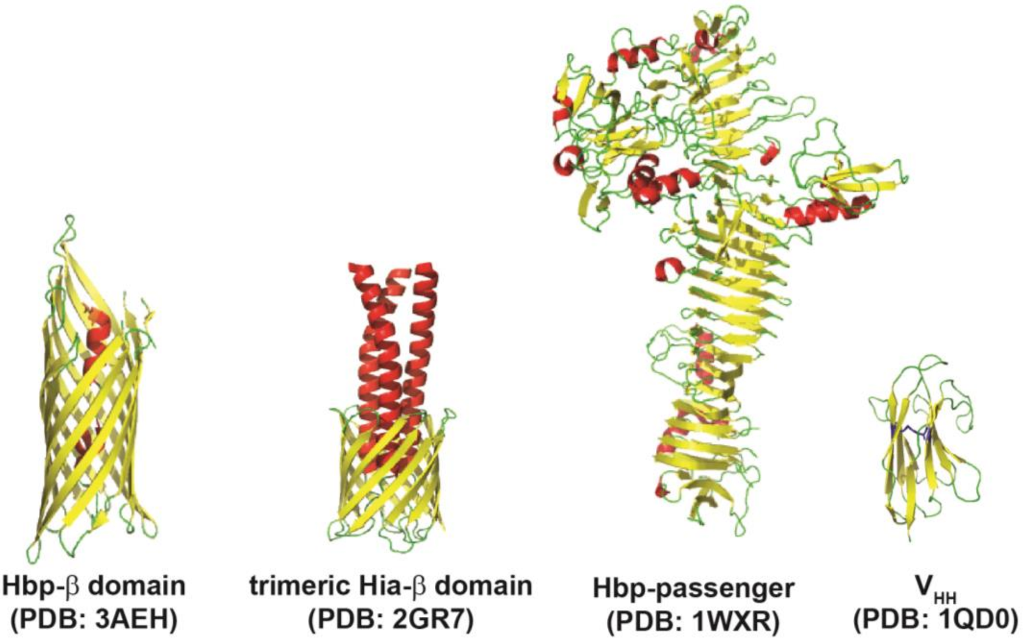

B Hiaβ

```
         SpeI
  1    actagtatta acggcagcca gttgtatgcc gtggcaaaag gggtaacaaa
          t  s  i   n  g  s   q  l  y  a   v  a  k   g  v  t

 51    ccttgctgga caagtgaata aagtgggcaa acgtgcagat gcaggtacag
       n  l  a  g   q  v  n   k  v  g   k  r  a  d   a  g  t

101    caagtgcatt agcggcttca cagttaccac aagcctctat gccaggtaaa
        a  s  a   l  a  a  s   q  l  p   q  a  s   m  p  g  k

151    tcaatggttt ctattgcggg aagtagttat caaggtcaaa gtggtttagc
          s  m  v   s  i  a   g  s  s  y   q  g  q   s  g  l

201    tatcggggta tcaagaattt ccgataatgg caaagtgatt attcgcttgt
       a  i  g  v   s  r  i   s  d  n   g  k  v  i   i  r  l

251    caggcacaac caatagtcaa ggtaaaacag gcgttgcagc tggtgttggt
        s  g  t   t  n  s  q   g  k  t   g  v  a   a  g  v  g
                    EcoRI
301    taccagtggt gagaattc
          y  q  w   -
```

C Hiaβ(3×)

```
       SpeI
  1   actagtatta acggcagcca gttgtatgcc gtggcaaaag gtgtaaccaa
        t  s  i   n  g  s   q  l  y  a   v  a  k   g  v  t

 51   ccttgctggt caagtgaata aagtgggcaa acgtgcagat gcaggtacgg
      n  l  a  g   q  v  n   k  v  g   k  r  a  d   a  g  t

101   caagtgcatt agcggcttca cagttaccac aagcctctat gccaggtaaa
       a  s  a   l  a  a  s   q  l  p   q  a  s   m  p  g  k

151   tcaatggttt ctattgcagg tagctcttat cagggtcaaa gtggtttagc
        s  m  v   s  i  a   g  s  s  y   q  g  q   s  g  l

201   tatcggggta tcacgtattt ccgataatgg caaagttatc atccgtttgt
      a  i  g  v   s  r  i   s  d  n   g  k  v  i   i  r  l

251   caggcaccac gaatagtcaa ggtaaaacgg gtgttgcagc tggtgttggt
       s  g  t   t  n  s  q   g  k  t   g  v  a   a  g  v  g

301   taccagtggg gcagcccggg taaaagcatg gtgtctattg cgggcagtag
        y  q  w   g  s  p   g  k  s  m   v  s  i   a  g  s

351   ctatcagggc caaagcggtc tggctatcgg cgtatctcgt atctccgata
      s  y  q  g   q  s  g   l  a  i   g  v  s  r   i  s  d

401   acggtaaagt tattatccgc ctgagcggca ccaccaactc tcagggcaaa
       n  g  k   v  i  i  r   l  s  g   t  t  n   s  q  g  k

451   accggtgtgg cagctggcgt tggttatcaa tggggttccc caggcaagtc
        t  g  v   a  a  g   v  g  y  q   w  g  s   p  g  k

501   aatggtctct atcgcgggca gcagttatca aggtcagagt ggcttagcga
      s  m  v  s   i  a  g   s  s  y   q  g  q  s   g  l  a

551   tcggtgtaag tcgtatttct gataacggca aagtgatcat tcgcctgagt
       i  g  v   s  r  i  s   d  n  g   k  v  i   i  r  l  s

601   ggcacgacca atagccaagg caaaacgggc gttgcggcag gtgtcggcta
        g  t  t   n  s  q   g  k  t  g   v  a  a   g  v  g
                 EcoRI
651   tcagtggtga gaattc
      y  q  w  -
```

S1 Fig. Structural models and nucleotide and protein sequence of Hiaβ and Hiaβ(3×). (A) Structural models of the Hbpβ, Hiaβ, Hbp passenger and V_{HH} domain. The PDB codes of the depicted models are given. **(B)** Nucleotide sequence of the *SpeI*-*Eco*RI fragment encoding the Hiaβ construct. Restriction sites used for cloning are indicated. The encoded amino acids are given below the nucleotide sequence with the amino acids constituting the four-stranded β-sheet contributing to the barrel in red. **(C)** Nucleotide sequence of the *SpeI*-*Eco*RI fragment encoding the Hiaβ(3×). The encoded amino acids are given below the nucleotide sequence with the amino acids constituting the three-times repeated segment in red, blue and pink.
