# Supplementary material for: Comparing autotransporter β-domain configurations for their capacity to secrete heterologous proteins to the cell surface
Source: PLoS One. 2018 Feb 7;13(2):e0191622. doi: 10.1371/journal.pone.0191622 (PMC5802855; doi:10.1371/journal.pone.0191622)
Supplement: S2 Fig — (A) Growth curves of cultures expressing the Hbp-β-domain fusions in MC10161 (left) and MC1061 degP::S210A (right). Expression was induced by adding IPTG at the timepoint indicated by the dotted line. (B) Western blots of cell samples of MC1061 expressing HbpΔβcleavage and Hbp-IgAPβ(1245) incubated with proteinase K either for 60 min on ice (0°) or 30 min at 37 °C (37°). Included are also untreated controls (-). A blot of a single SDS-PAGE gel was cut in two parts after which the top part was incubated with α-SurA antiserumem and the bottom part with α-OmpA antiserum. (PDF) [file pone.0191622.s002.pdf]

A

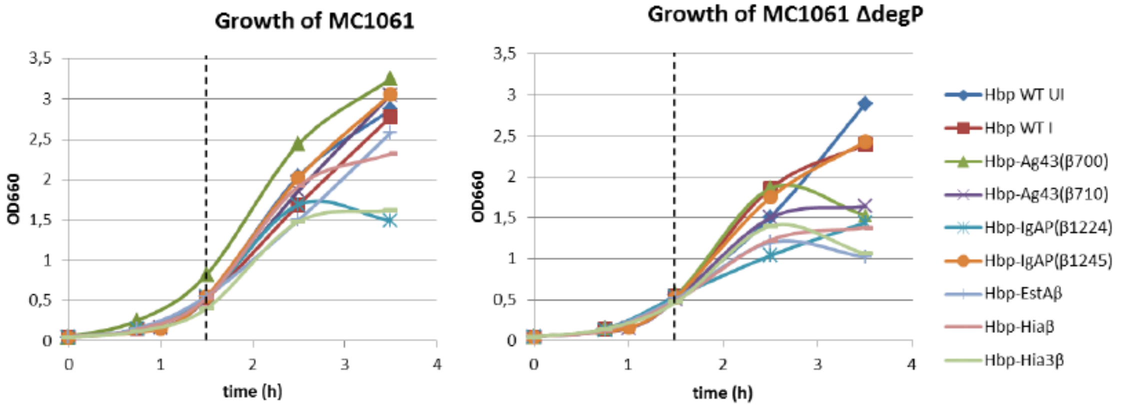

B

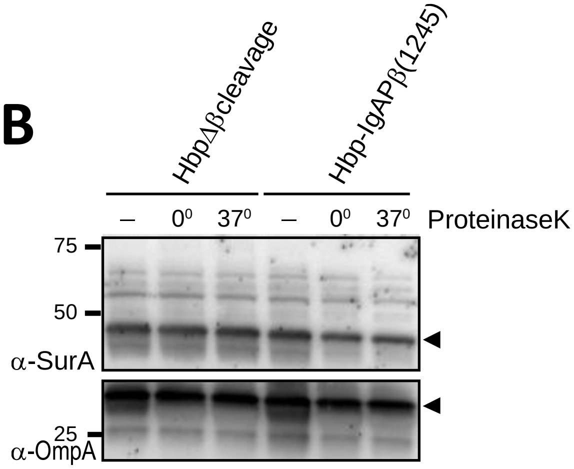

S2 Fig. Expression of the Hbp-β-domain fusions. (A) Growth curves of cultures expressing the Hbp-β-domain fusions in MC10161 (left) and MC1061 *degP*::S210A (right). Expression was induced by adding IPTG at the timepoint indicated by the dotted line. (**B)** Western blots of cell samples of MC1061 expressing HbpΔβcleavage and Hbp-IgAPβ(1245) incubated with proteinase K either for 60 min on ice (0°) or 30 min at 37 °C (37°). Included are also untreated controls (-). A blot of a single SDS-PAGE gel was cut in two parts after which the top part was incubated with α-SurA antiserumem and the bottom part with α-OmpA antiserum.
