# Supplementary material for: Comparing autotransporter β-domain configurations for their capacity to secrete heterologous proteins to the cell surface
Source: PLoS One. 2018 Feb 7;13(2):e0191622. doi: 10.1371/journal.pone.0191622 (PMC5802855; doi:10.1371/journal.pone.0191622)
Supplement: S3 Fig — (A) Growth curves of cultures expressing the VHH-β-domain fusions in in MC10161 (top left), MC1061 degP::S210A (top right), DHB4 (bottom left) and DHBA (dsbA-; bottom right). Expression was induced by adding IPTG at the timepoint indicated by the dotted line. (B) Western blots of cell samples in DHB4 and DHBA (dsbA-) cells. Westernblots incubated with α -Myc of whole cell lysates and culture supernatants of cultures of DHB4 (left panel) and DHBA (dsbA-) (right panel) expressing the VHH-β-domain fusions (*) to assess their expression. (C) Western blot of MC1061 cells expressing VHH-Hbpβ and VHH-IgAPβ(1245) incubated with proteinase K either for 30 min at 37°C (37°). Included are also untreated controls (-). A blot of a single SDS-PAGE gel was cut in two parts after which the top part was incubated with α-SurA antiserum and the bottom part with α-OmpA antiserum. The relevant lanes were cut from a larger image to place them side by side. (PDF) [file pone.0191622.s003.pdf]

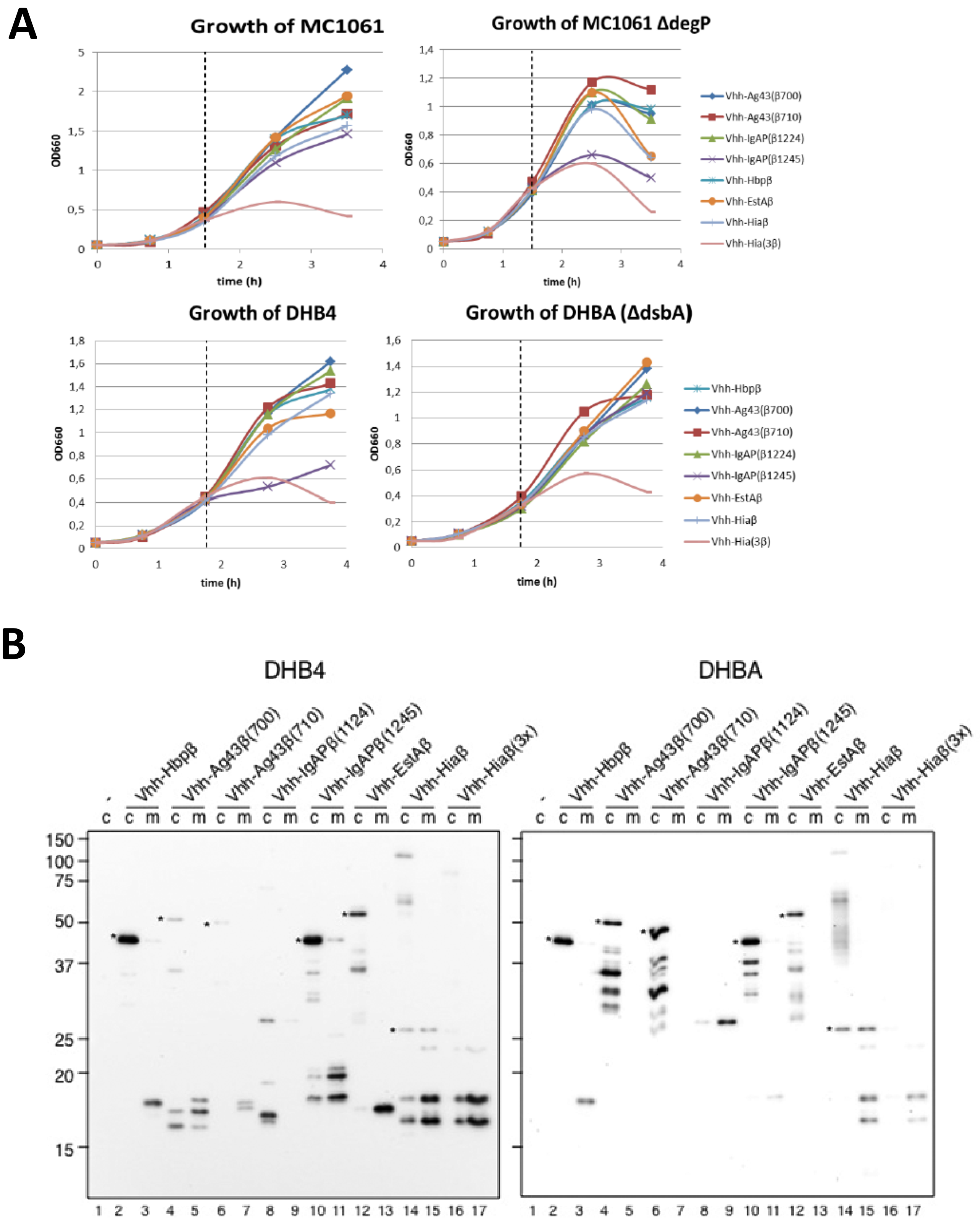
A
Growth of MC1061
Growth of MC1061 ΔdegP
Growth of DHB4
Growth of DHBA (ΔdsbA)
OD660
time (h)
Vhh-Ag43(β700)
Vhh-Ag43(β710)
Vhh-IgAP(β1224)
Vhh-IgAP(β1245)
Vhh-Hbpβ
Vhh-EstAβ
Vhh-Hiaβ
Vhh-Hia(3β)
B
DHB4
DHBA
Vhh-Hbpβ
Vhh-Ag43β(700)
Vhh-Ag43β(710)
Vhh-IgAPβ(1124)
Vhh-IgAPβ(1245)
Vhh-EstAβ
Vhh-Hiaβ
Vhh-Hiaβ(3x)
c c m c m c m c m c m c m c m c m
150
100
75
50
37
25
20
15
1 2 3 4 5 6 7 8 9 10 11 12 13 14 15 16 17

C

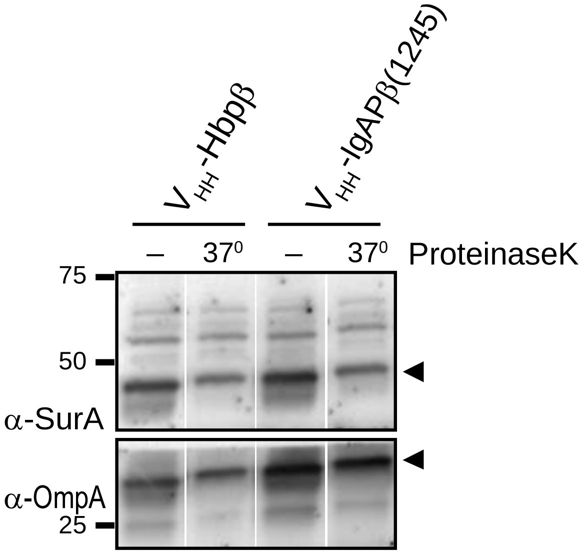

S3 Fig. Expression of V_{HH}-β-domain fusions. (A) Growth curves of cultures expressing the V_{HH}-β-domain fusions in in MC10161 (top left), MC1061 *degP*::S210A (top right), DHB4 (bottom left) and DHBA (*dsbA*$^{-}$; bottom right). Expression was induced by adding IPTG at the timepoint indicated by the dotted line. **(B)** Western blots of cell samples in DHB4 and DHBA (*dsbA*$^{-}$*)*cells. Westernblots incubated with α -Myc of whole cell lysates and culture supernatants of cultures of DHB4 (left panel) and DHBA (*dsbA*$^{-}$) (right panel) expressing the V_{HH}-β-domain fusions (∗) to assess their expression. **(C)** Western blot of MC1061 cells expressing V_{HH}-Hbpβ and V_{HH}-IgAPβ(1245) incubated with proteinase K either for 30 min at 37°C (37°). Included are also untreated controls (-). A blot of a single SDS-PAGE gel was cut in two parts after which the top part was incubated with α-SurA antiserum and the bottom part with α-OmpA antiserum. The relevant lanes were cut from a larger image to place them side by side.
