# Supplementary material for: Comparing autotransporter β-domain configurations for their capacity to secrete heterologous proteins to the cell surface
Source: PLoS One. 2018 Feb 7;13(2):e0191622. doi: 10.1371/journal.pone.0191622 (PMC5802855; doi:10.1371/journal.pone.0191622)
Supplement: S1 Table — (PDF) [file pone.0191622.s004.pdf]

S1 Table. Primer sequences used for cloning the β-domain fusions.

Primer name	*Primer sequence*
Hbp 2734 fw	cagatgttgtacagcctg
Hbp SpeI fw	cattcatgcac**actagt**tataacaacttc
Hbp SpeI rv	gaagttgttata**actagt**gtgcatgaatG
EcoR1 Hbp rv	gatcgaattctcagaatgaataacgaatattagc
SpeI-Ag43-β-short fw	tagtca**actagt**gtccccctgtatgcctccatg
SpeI-Ag43-β-long fw	tagtca**actagt**ctgcgcagtgaaaatgcttatc
Ag43-β rv	ctga**gaattc**tcagaaggtcacattcagtgtgg
SpeI-IgAP-β short fw	tagtca**actagt**accaatactaactcggctttatc
SpeI-IgAP-β-long fw	tagtca**actagt**gtattttcattggatgattatgatg
IgAP-β rv	ctga**gaattc**tcagaaacgaatctgtattttaatttgtc
SpeI-EstA-β fw	tagtca**actagt**Ccgaccatcaccggccag
EstA-β rv	ctga**gaattc**tcagaagtccaggctcagcgc
SpeI-Hia-β fw	tagtca**actagt**attaacggcagccagttgtatg
Hia-β rv	ctga**gaattc**tcaccactggtaaccaacaccag
pEH XbaI-Hbp fw	taactttctagattacaaaacttaggagggtttttaccatgaacag aatttattctcttcg
VHH(R2)-GSGSG-SpeI rv	tagtca**actagt**accgctgccggatcccagatc

*Spe*I (actagt) and EcoRI (gaattc) restriction sites in bold.
